# Highly effective and chemically stable surface enhanced Raman scattering substrates with flower-like 3D Ag-Au hetero-nanostructures

**DOI:** 10.1038/s41598-018-19165-9

**Published:** 2018-01-17

**Authors:** Ying Zhang, Chengliang Yang, Bin Xue, Zenghui Peng, Zhaoliang Cao, Quanquan Mu, Li Xuan

**Affiliations:** 1 State Key Laboratory of Applied Optics, Changchun Institute of Optics, Fine Mechanics and Physics, Chinese Academy of Sciences, Changchun, Jilin, 130033 China; 2 University of Chinese Academy of Sciences, Beijing, 100049 China; 3 State Key Laboratory of Luminescence and Applications, Changchun Institute of Optics, Fine Mechanics and Physics, Chinese Academy of Sciences, Changchun, Jilin, 130033 China

## Abstract

We demonstrated flower-like 3D Ag-Au hetero-nanostructures on an indium tin oxide glass (ITO glass) for surface enhanced Raman scattering (SERS) applications. The flower-like 3D Ag nanostructures were obtained through electrodeposition with liquid crystalline soft template which is simple, controllable and cost effective. The flower-like 3D Ag-Au hetero-nanostructures were further fabricated by galvanic replacement reaction of gold (III) chloride trihydrate (HAuCl_4_·3H_2_O) solution and flower-like Ag. The flower-like Ag-Au hetero-nanostructure exhibited stronger SERS effects and better chemical stability compared with flower-like Ag nanostructure. The localized surface plasmon resonance (LSPR) spectra, field emission scanning electron microscope (FESEM) photos and Ag-Au ratios were studied which show that the surface morphology and shape of the flower-like Ag-Au hetero-nanostructure play significant roles in enhancing SERS. The flower-like 3D Ag-Au hetero-nanostructures fabricated by electrodeposition in liquid crystalline template and galvanic replacement reaction are simple, cheap, controllable and chemical stable. It is a good candidate for applications in SERS detection and imaging.

## Introduction

Surface enhanced Raman scattering (SERS) is a technique for enhancing the Raman scattering intensity which rapidly becomes a powerful method for applications in fundamental and applied research such as medicine^1–3^, biotechnology^4–6^, catalysis^7^, imaging^8^ and sensing^9–11^. One of the most active research aspects in SERS is the seeking for substrates with property of improved enhancement factor, good reproducibility, fabrication simplicity, controllability, good chemical stability and low cost. As known to all, silver nanostructures embrace SERS properties with large enhancement at visible-excitation wavelengths for highly sensitive detection of chemical or biological species^12–14^. However, elemental silver is easily to be oxidized in the atmosphere especially with halide ions, acid, UV irradiation and heating^15–19^. The metal nanostructures in small size (nm) with high surface free energies lead to poor chemical stability^20^ and significantly reduce their enhancement effect which limits its further applications. It’s necessary to improve the stability of nanostructure substrate as well as the enhancement ability. Metal nanostructures with high enhancement factor and good chemical stability are one of the focuses in SERS research. The effective solution for improving the chemical stability of silver nanostructures is to form an alloy or hetero-nanostructures with a more stable metal such as Au^15,21^. However, such method may reduce the SERS enhancement effect because the electrons in Au are less sensitive to the electric field^22^. The study of reaction conditions for Ag-Au nanostructures with the best enhancement effect is still needed. For improved enhancement factor, it is found that 3D nanostructures such as flower-like structures used as SERS substrates exhibit more favorable properties such as high enhancement factor owing to more ‘hotspots’ and enhanced local EM field than lower dimensional nanostructures^23–25^. The flower-like metal nanostructure substrates with high enhancement effects show considerable advantages in SERS field. However, there are still some problems with the fabrication of flower-like metal nanostructures such as complex production process, high cost, time consuming, poor reproducibility and so on, which limit the practical applications for the substrates. The flower-like metal nanostructure substrates with large scale uniformity, high enhancement factor and high chemical stability still need to be studied.

In this work, we investigated the flower-like Ag-Au hetero-nanostructures on an ITO glass substrate for SERS applications by electrodeposition with liquid crystal template and galvanic replacement reaction which is simple and cost-effective. The SERS enhancement factor of 1.17 × 10^7^ was achieved with the flower-like silver nanostructures. Different flower-like Ag-Au hetero-nanostructures were obtained for different concentrations of HAuCl_4_ and different reaction times based on the flower-like silver nanostructures. The enhancement factor of 8.6 × 10^7^ was achieved by the flower-like Ag-Au hetero-nanostructures. The reasons for the changes of enhancement factors with different Ag-Au nanostructures were studied. The chemical stability was also studied which shows that the flower-like Ag-Au hetero-nanostructures is better than the flower-like Ag nanostructures. Such Ag-Au hetero-nanostructures fabricated by liquid crystal template and galvanic replacement reaction are good candidate for applications in SERS detection and imaging.

## Results and Discussion

### Morphology and constitute characterization

**Figure 1:**
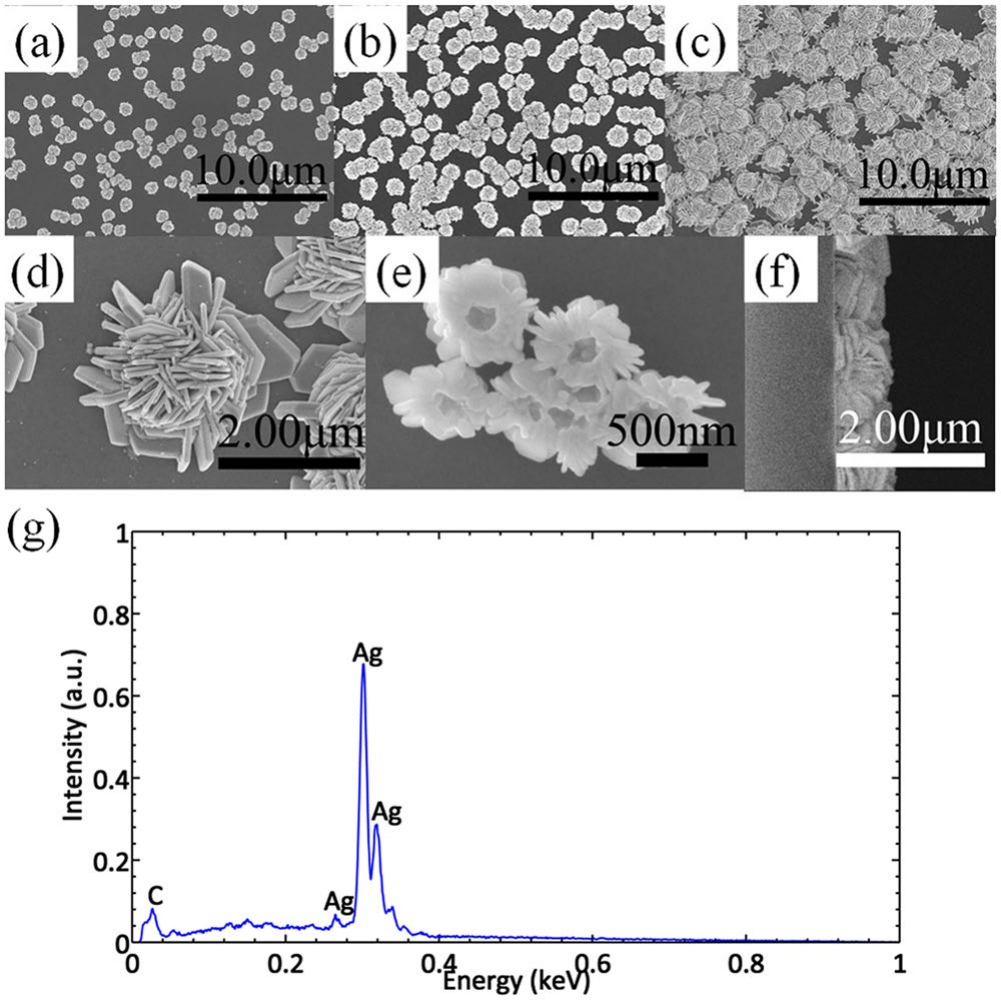
Typical characterizations of flower-like silver nanostructure substrates. (**a–c**) Are FESEM pictures of silver flower nanostructure substrates obtained for 0.5 h, 2 h and 5 h, respectively. (**d**) Is the top view of single silver flower. (**e**) Is the image from the bottom of silver flowers. (**f**) is the side view of the nanostructure substrate. (**g**) Is EDS spectrum of sliver flowers.

In this work, we used electrodeposition method with the liquid crystalline phase as the soft template to fabricate the flower-like silver nanostructures reported by us in previous work with a little difference^26^. In the electrodeposition process, the liquid crystalline phase was used as template which controls the formation of nanostructures. Different flower-like silver nanostructures were obtained for 0.5 h, 2 h and 5 h deposition time. The morphology was observed by a field emission scanning electron microscope (FESEM). The chemical constituent of the nanostructure was characterized by the energy dispersive spectrum (EDS). Figure 1 shows the images of flower-like silver nanostructures obtained for different growth times and the energy dispersive spectrum (EDS). Figure 1(a–c) are the FESEM images of the silver flowers for 0.5 h, 2 h and 5 h respectively, from which we can see that both of the coverage ratio and size of silver flowers increase with growth time. Figure 1(d–f) are magnified images of silver flowers which are taken from different directions. Figure 1(d) is the top view of single silver flower (5 h). The structure of the fabricated silver nanostructure is quite like a flower composed of petals whose thickness is about 50 nm. Between the petals there are many horns and thin gaps. Figure 1(e) is the image from the bottom of silver flowers (0.5 h). From the picture we can see that there is a round hole in the center of each silver flower. The flower-like silver nanostructures are hollow which is good for SERS effect^15^. Figure 1(f) is the side view of the nanostructures (5 h). The height of the flowers is about 700 nm. The chemical constituent of the flowers is shown in Fig. 1(g). The EDS result shows that the dominant peak is for the elemental silver which indicates that the flower-like nanostructures are mainly composed of metallic silver. The weak peaks for carbon and other elements can be attributed to the residue liquid crystal template.

**Figure 2:**
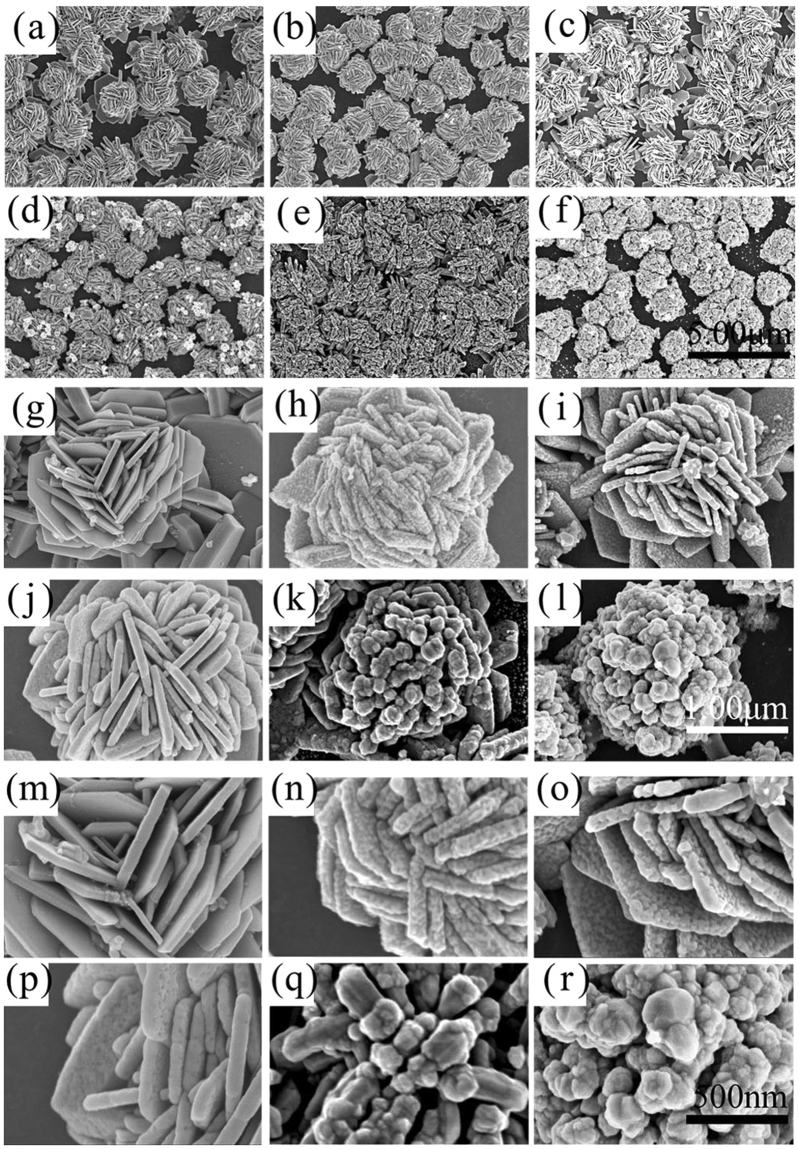
FESEM images of flower-like Ag nanostructures and flower-like Ag-Au hetero-nanostructures. (**a**) Is the image of silver flower before reacting with the HAuCl_4_. **(b–d)** Are images of flower-like Ag-Au hetero-nanostructures obtained by reacting with 0.05 mM aqueous solutions of HAuCl_4_ for 10 min, 30 min and 40 min respectively. (**e–f**) Are images of flower-like Ag-Au hetero-nanostructures obtained by reacting with 0.5 mM aqueous solutions of HAuCl_4_ for 10 min and 30 min respectively. (**g–l**) Are the single flower images of (**a–f**). (**m–r**) Are the magnified images of (**g–l**).

The galvanic replacement reaction between elemental Ag and HAuCl_4_ was performed to synthesis the flower-like Ag-Au hetero-nanostructures. We chose the flower-like silver nanostructures for 5 h growth time to react with HAuCl_4_. The flower-like Ag-Au hetero-nanostructures with different morphologies were obtained by taking Ag flower to react with different concentration of HAuCl_4_ for different times. Figure 2(a) is the image of silver flower (5 h) before reacting with the HAuCl_4_. Figure 2(b–f) are the FESEM images with low magnification of the flower-like Ag-Au hetero-nanostructures. Figure 2(b–d) are images of flower-like Ag-Au hetero-nanostructures obtained by reacting with 0.05 mM aqueous solutions of HAuCl_4_ for 10 min, 30 min and 40 min respectively. Figure 2(e–f) are images of flower-like Ag-Au hetero-nanostructures obtained by reacting with 0.5 mM aqueous solutions of HAuCl_4_ for 10 min and 30 min respectively. Figure 2(g–l) are the single flowers’ images of (a–f), respectively. Figure 2(m–r) are the magnified images of (g–l). From the SEM pictures, we see that the morphologies of flower-like Ag-Au hetero-nanostructures vary with different reaction conditions such as concentration of HAuCl_4_ and reaction time. When HAuCl_4_ solution is in low concentration (0.05 mM), the global shapes of the nanostructures do not change while some decorations of Au nanoparticles are added on the surface of the flower-like silver nanostructures. Furthermore, the sizes of each Au nanoparticles become larger as the reaction time increases (diameter of 13 nm to 25 nm with reaction time from 10 min to 30 min). As the reaction time increasing, the Au nanoparticles form a film with some dishes whose diameters are about 25 nm (Fig. 2(j,p)). When HAuCl_4_ solution is in high concentration (0.5 mM), the global shapes of the nanostructures are changed. The petals of flower-like silver nanostructures are capped with Au atoms and are also cut off partly owing to the loss of Ag in the replacement reaction (one Au atom replaces three Ag atoms) when the reaction time is 10 min. When the reaction time is 30 min, some big Ag@Au nanoparticles make up the flower-like Ag-Au hetero-nanostructures.

**Figure 3:**
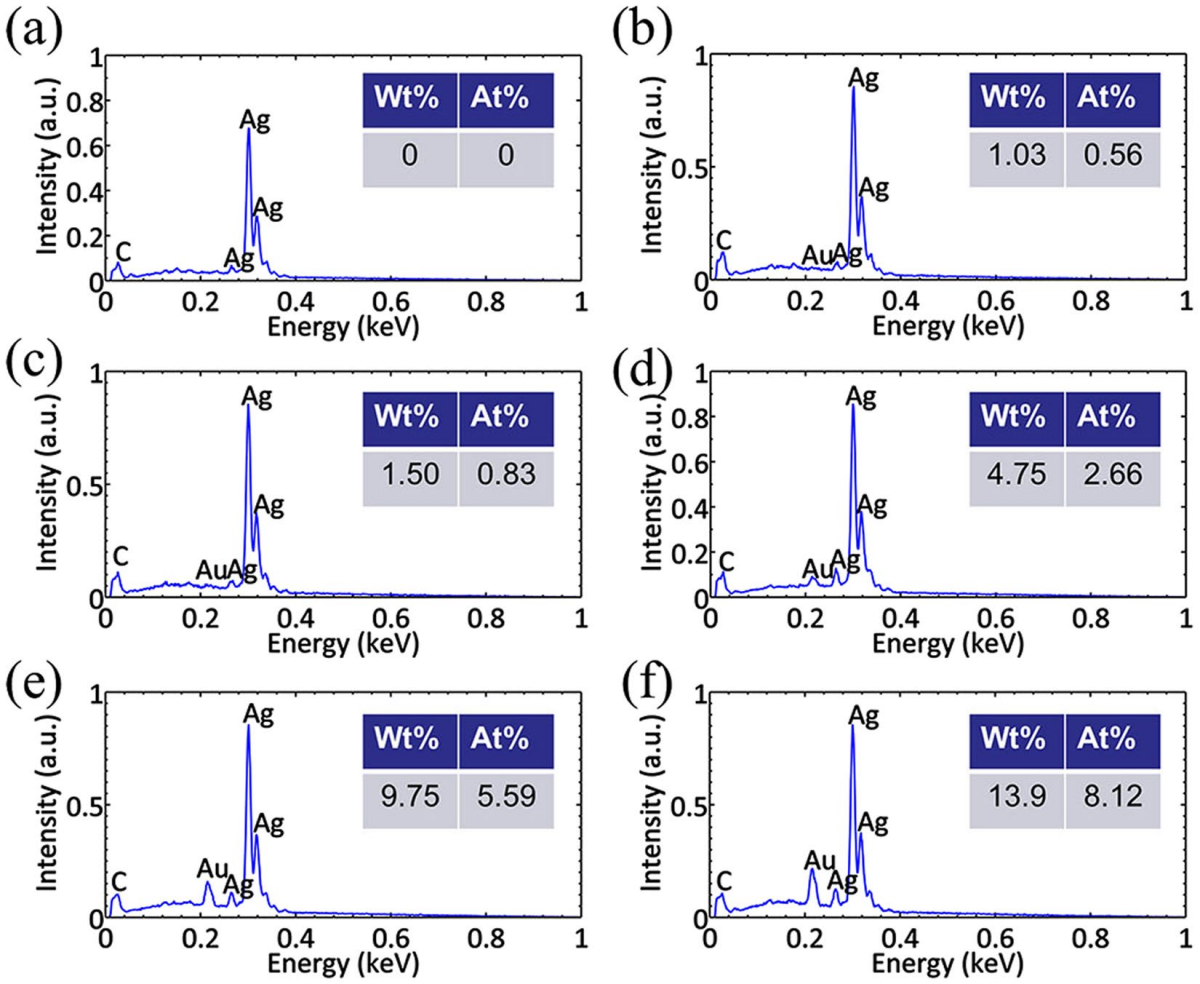
EDS spectra of Ag nanostructures and Ag-Au hetero-nanostructures. (**a**) Is the spectrum of silver flower before reacting with the HAuCl_4_. (**b–d**) Are spectra of Ag-Au hetero-nanostructures obtained by reacting with 0.05 mM aqueous solutions of HAuCl_4_ for 10 min, 30 min and 40 min respectively. **(e–f)** Are spectra of Ag-Au hetero-nanostructures obtained by reacting with 0.5 mM aqueous solutions of HAuCl_4_ for 10 min and 30 min respectively. The inserted tables are the Au:AuAg ratios in the different nanostructures.

The chemical constituent of the flower-like Ag-Au hetero-nanostructures are analyzed by the EDS spectra (Fig. 3). Figure 3(a) is the EDS spectrum of flower-like silver nanostructure before reacting with HAuCl_4_, from which we can see that there is no Au element in this structure. Figure 3(b–d) are the EDS spectra of Ag-Au hetero-nanostructures obtained by reacting with 0.05 mM aqueous solutions of HAuCl_4_ for 10 min, 30 min and 40 min respectively and (e,f) are the EDS spectra of Ag-Au hetero -nanostructures obtained by reacting with 0.5 mM aqueous solutions of HAuCl_4_ for 10 min and 30 min respectively. The inserted tables show the Au:AuAg ratios in the different flower-like Ag-Au hetero-nanostructures. The Au:Ag ratio rises as the concentration of Au^3+^ in the reaction solution increases. Increased Au:AuAg ratios in the formed Ag-Au hetero-nanostructures are observed.

### LSPR properties

Both Ag and Au show particular localized surface plasmon resonance properties dependent on the morphology and structures^27^. As we know, scattering and absorption will occur when the beam goes through the scattering body. The LSPR spectrum which is usually used to characterize the LSPR properties can be calculated from 1 minus the transmission (thus scattering and absorption). In the LSPR spectra measurement, we use the Perkin Elmer Lambda 950 spectrophotometer with an integrating sphere to characterize the LSPR properties of the nanostructures. Firstly, we measured the intensity of the spectrum without sample which was denoted as I. Then, the sample was vertically placed to the light. The intensity of the spectrum with sample was measured which was denoted as I’. Then the transmittance was obtained by I’/I. Considering the influence of ITO glass on the transmittance, we measured the transmittance of the ITO glass without silver nanostructures which was used as the reference. By rotating the monochromator the whole LSPR spectrum can be obtained. The LSPR spectra of flower-like silver nanoparticles fabricated for 5 h and different flower-like Ag-Au hetero-nanostructures obtained by reaction with HAuCl_4_ were obtained.

**Figure 4:**
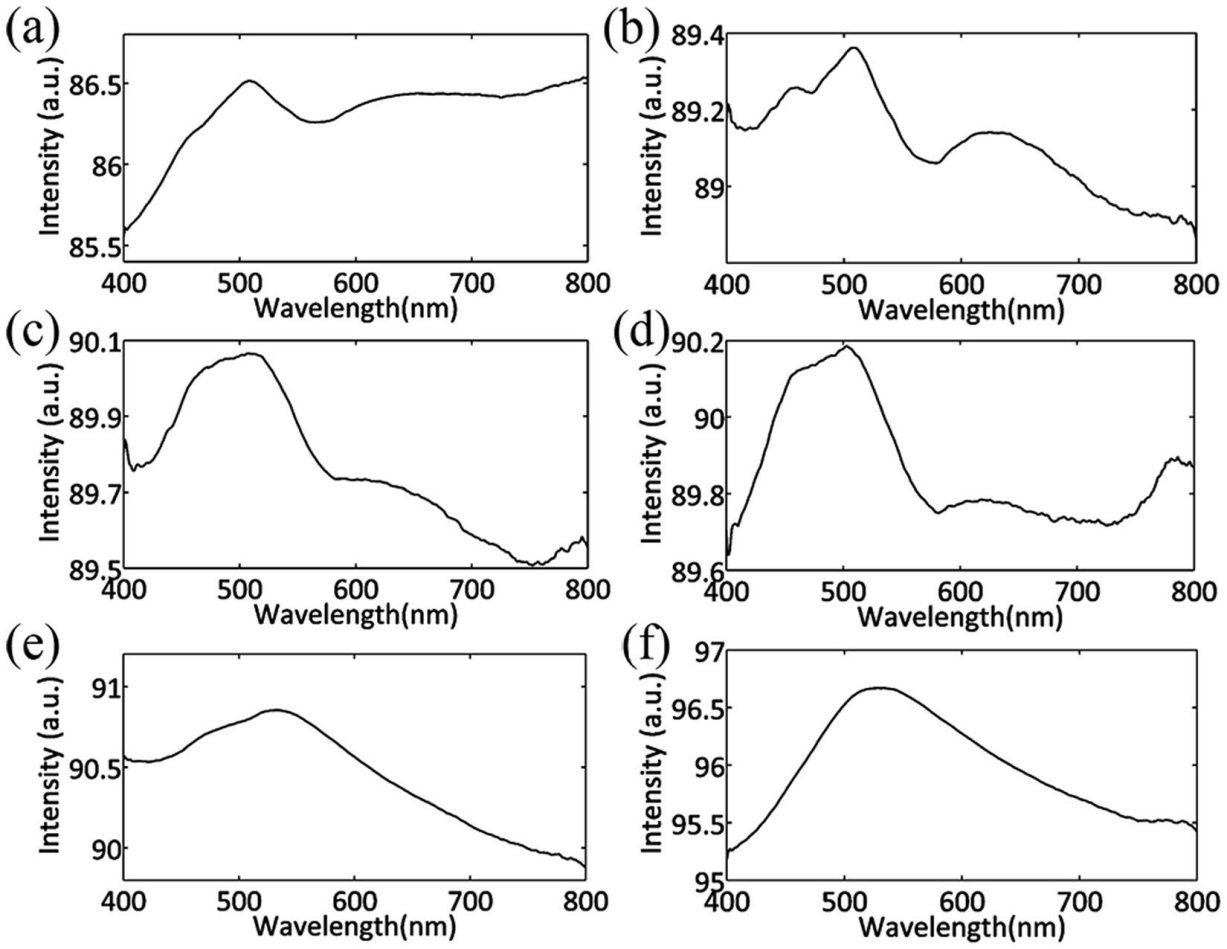
LSPR spectra of flower-like Ag nanostructures and flower-like Ag-Au hetero-nanostructures. (**a**) Is the spectrum of flower-like silver nanostructures before reacting with the HAuCl_4_. (**b–d**) Are spectra of Ag-Au hetero -nanostructures obtained by reacting with 0.05 mM aqueous solutions of HAuCl_4_ for 10 min, 30 min and 40 min respectively. (**e–f**) Are spectra of Ag-Au hetero-nanostructures obtained by reacting with 0.5 mM aqueous solutions of HAuCl_4_ for 10 min and 30 min respectively.

Figure 4 shows the LSPR spectra for flower-like silver nanostructure for 5 h growth time (Fig. 4(a)), the Ag-Au hetero-structures obtained by reaction with 0.05 mM HAuCl_4_ for 10 min (Fig. 4(b)), 0.05 mM HAuCl_4_ for 30 min (Fig. 4(c)), 0.05 mM HAuCl_4_ for 40 min (Fig. 4(d)), 0.5 mM HAuCl_4_ for 10 min (Fig. 4 (e)), 0.5 mM HAuCl_4_ for 30 min (Fig. 4(f)). The LSPR bands are appeared at 458 nm, 508 nm and 650 nm for flower-like silver nanostructures. For Ag-Au hetero-nanostructures obtained by reaction with 0.05 mM HAuCl_4_, the bands at 458 nm and 508 nm are not changed, while the 650 nm peak are blue shifted to 624 nm. The intensities of the bands are enhanced with the reaction time increasing. For the Ag-Au hetero-nanostructures obtained by reaction with 0.5 mM HAuCl_4_, the shapes were strongly changed. When the reaction time is 10 min, the number of the bands changes from 3 to 2 which are located at 473 nm and 530 nm. They are all red-shifted compared with the corresponding bands at 458 nm and 504 nm for silver flower. As the reaction time increases to 30 min, the number of the bands changes from 2 to 1 located at 527 nm. Combining the SEM photos, the lower-order multipole resonance is more sensitive to the morphology changes than high-order multipole resonance (Fig. 4(a–d)). When the total shapes of the nanostructures change a lot, there are changes both of lower-order multipole resonance and high-order multipole resonance (Fig. 4(e–f)). Because of the fact that the LSPR band of pure Au nanoparticles for tens to 100 nm is almost located at around 528 nm^28^, the bands of Ag-Au nanostructures are getting closer to 528 nm (Fig. 4(f)) with the Au increasing. The intensities of spectra are enhanced with the Au ratio increasing. Comparing the SEM photos, the total shapes of the Ag-Au hetero-nanostructures obtained by reaction with 0.05 mM HAuCl_4_ do not change largely while the surface morphology obviously change shown as Fig. 2(a–d). The bands which correspond to lower-order plasmon resonances such as dipole resonance mode and quadrupole resonance mode in the LSPR spectra are sensitive to the morphology changes (Fig. 4(a–d)). The shapes of the Ag-Au hetero-nanostructures obtained by reaction with 0.5 mM HAuCl_4_ change largely (Fig. 2(e–f)) leading to the changes of the bands which correspond both to higher-order plasmon resonances modes and the lower-order plasmon resonances in the LSPR spectra (Fig. 4(e–f)). With the quantities of Au in the Ag-Au nanostructures increasing, the resonance bands are getting closer to 528 nm and the intensities of the LSPR spectra gradually increase. The intensities of Fig. 4(a–f) are intensified at 785 nm.

### Surface enhanced Raman Scattering effects

**Figure 5:**
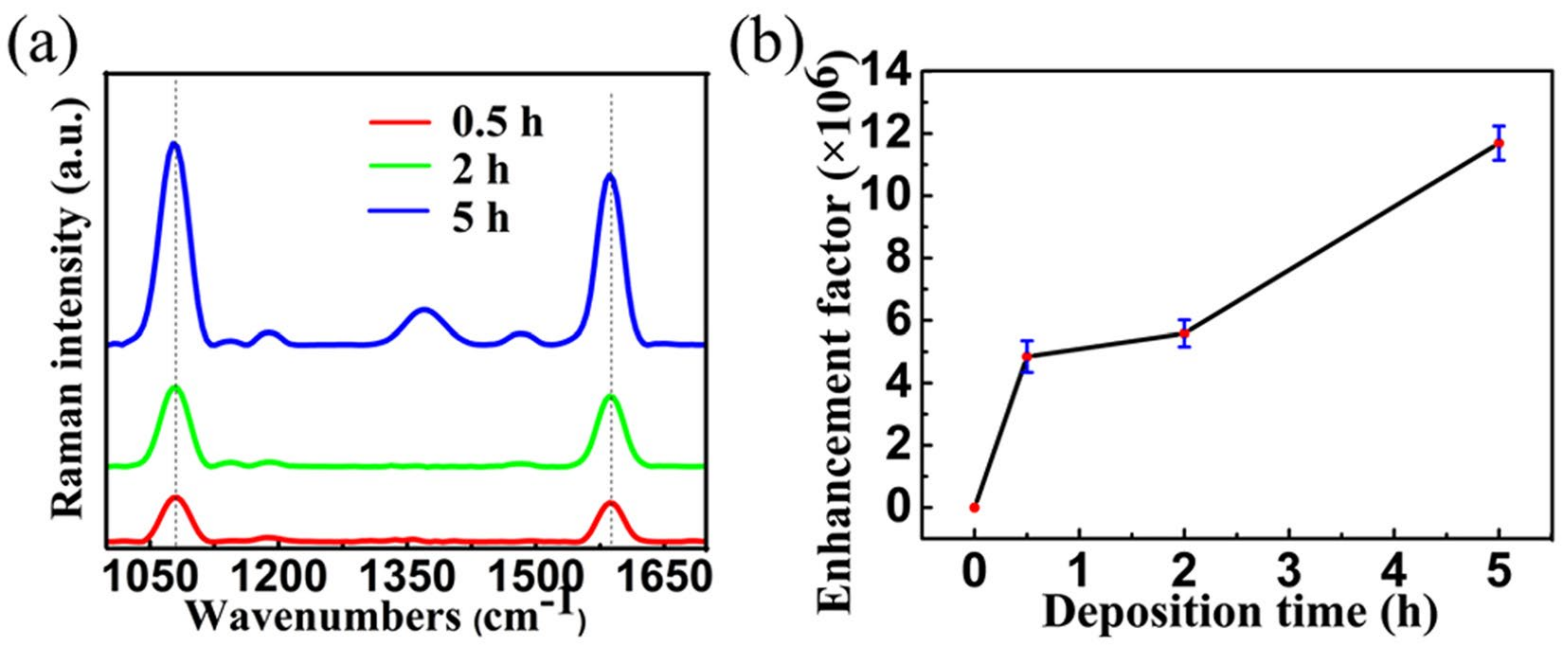
SERS effect for flower-like silver nanostructure substrates with different growth time. (**a**) SERS spectra of 4-MBA for substrates of 0.5 h, 2 h, 5 h deposition time respectively. (**b**) Is the relation between enhancement factor and deposition time.

We studied the SERS properties of flower-like silver nanostructure substrates with 0.5 h, 2 h and 5 h deposition time. The Ag flowers were immersed in a 50 μM aqueous solution of 4-MBA overnight followed by the washing of deionized water. 3 μL aqueous solution of 4-MBA (500 mM) was dropped on a silicon wafer as reference sample. Figure 5(a) shows the SERS spectra of 4-mercaptobenzoic acid (4-MBA) molecules adsorbed on the surface of flower-like silver nanostructures with excitation wavelength of 785 nm. Two obvious characteristic peaks of 1080 cm^−1^ and 1586 cm^−1^ of 4-MBA are shown in Fig. 5(a). The intensities of peaks are enhanced by the flower-like silver nanostructure substrates and increase with the deposition time increasing. To evaluate the enhancement, we calculated the SERS enhancement factors (EFs) with a conventional method^29–34^ using the following formula^35^: $EF=({I}_{SERS}/{I}_{Raman})\times ({N}_{Raman}/{N}_{SERS})$, where *I*_*SERS*_ and *I*_*Raman*_ denote the intensities of SERS and normal Raman spectra, respectively, *N*_*Raman*_ and *N*_*SERS*_ are numbers of molecules for normal Raman measurement and SERS measurement, respectively. Based on the intensity of the peak at ∼1080 cm^−1^, the SERS EFs (details are in the Supplementary Information) were evaluated as 4.84 × 10^6^, 5.58 × 10^6^ and 1.17 × 10^7^ for flower-like silver nanostructures with 0.5 h, 2 h and 5 h deposition time, respectively.

**Figure 6:**
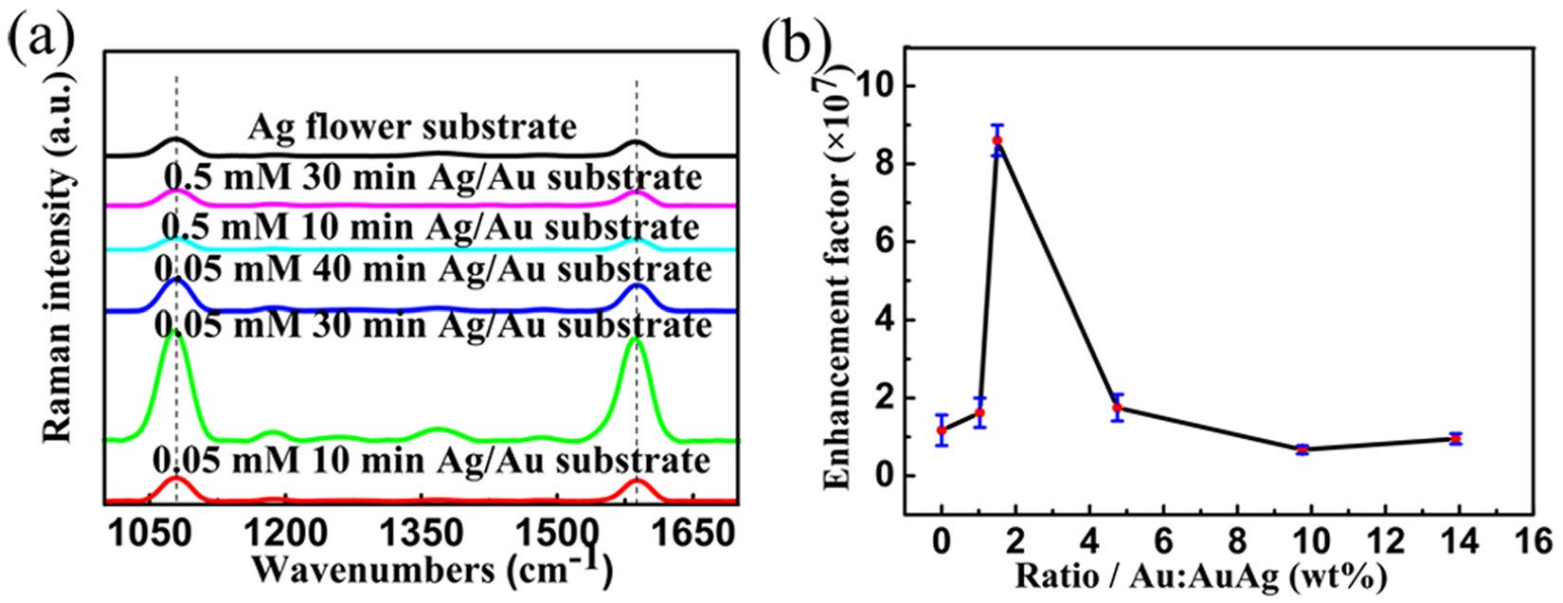
SERS effects of Ag-Au hetero-nanostructure substrates with different reaction time compared with SERS effect of silver nanostructure substrate. (**a**) SERS spectra and Raman spectra of 4-MBA for flower-like Ag-Au hetero-nanostructure substrates and flower-like silver nanostructure substrate respectively. (**b**) Is the relation between enhancement factor and gold ratio in the hetero-nanostructure substrate.

Flower-like Ag-Au hetero-nanostructures were fabricated by the galvanic replacement reaction with the HAuCl_4_ solution based on the flower-like silver nanostructure obtained for 5 h whose EF is the largest from the experiments above. SERS spectra from flower-like Ag-Au hetero-nanostructure substrates obtained by different reaction time were also collected which are shown in Fig. 6(a). Due to the existence of Au^3+^ from the reaction, the intensity of the SERS peaks changes a lot. In order to evaluate the enhancement effects by different substrates, EFs are calculated by the method mentioned above based on the intensity of the peak at ~1080 cm^−1^. The EFs are 1.62 × 10^7^, 8.6 × 10^7^, 1.75 × 10^7^, 6.7 × 10^6^ and 9.5 × 10^6^ for Ag-Au hetero-nanostructure substrates obtained by reacting with 0.05 mM aqueous solutions of HAuCl_4_ for 10 min, 30 min and 40 min and 0.5 mM for 10 min and 30 min, respectively.

The SERS properties for the flower-like Ag nanostructures and flower-like Ag-Au hetero-nanostructures are different obviously. The enhancement factors increase firstly then decrease with the increasing of Au ratio (Fig. 6(b)). We analyzed the reasons for enhancement of Raman signal combing SEM photos with the LSPR spectra in the next part.

### Analysis

Based on the model of harmonic oscillator of Raman scattering, the intensity of the Raman scattering is influenced by the change of polarizability $\partial \alpha /\partial {q}_{0}$ and the intensity of excitation electric field ${\overrightarrow{E}}_{0}$. The intensity of the Raman scattering can be enhanced by improving the values of the $\partial \alpha /\partial {q}_{0}$ and ${\overrightarrow{E}}_{0}$, which is the theory of surface enhanced Raman scattering as chemical enhancement and electromagnetic enhancement. The enhancement factor induced by chemical enhancement is also 10–10^3^^36^, while electromagnetic enhancement is major factor in surface enhanced Raman scattering. As we all know, the Raman enhancement effect is directly proportional to $|E{|}^{4}/|{E}_{0}{|}^{4}$^37^, where *E* is localized electric field and *E*_0_ is the excitation electric field. The value of the electric field $E/{E}_{0}$ can be used as the criterion to evaluate the SERS effect of the metal nanostructures which is derive from surface localized electric field enhancement on the surface of metal nanostructures^38,39^.

Surface localized electric field enhancement can be accounted by the models of localized surface plasmon resonance and lightning rod effect. When the electromagnetic wave goes to the surface of the metal nanostructures, the collective electron oscillated. The localized surface plasmon resonance is occurred when the frequency of the electromagnetic is equal to the frequency of the oscillated electron. The localized electric filed is largely enhanced. Based on the lightning rod effect, horns and thin gaps are “hot spots”, where the intensity of the electric field are large. The horns can focus the energy in a small volume which lead to large electric field enhancement. The charges located on the both sides of the thin gaps are increased based on the phase retard effect leading to the surface electric field enhancement. Above all, the ultimate effects of both the models of the localized surface plasmon resonance and lightning rod effect are the localized electric filed enhancement.

For the flower-like nanostructures we fabricated, there are a lot of “hot spots”, which verifies that the lightning rod effects on these structures play important roles in the high surface enhanced Raman scattering. Combining the LSPR spectra and SEM photos of the nanostructures, according to the discrete dipole approximation theory, the roughened flower-like silver nanostructure can be divided into a large core particle and small peripheral particles. Therefore, the LSPR properties can be attributed to multipole resonance of the core particle and dipole resonance of peripheral particles. The overall electric field equals to the sum of radiation field from the core particle and peripheral particles. Besides, the coherent superposition of the dipolar plasmon radiation fields originated from different peripheral particles can be occurred which leads to amplified electric field. In like manner, for the Ag-Au hetero-nanostructures, the coherent superposition of the dipolar plasmon radiation fields originated from Au nanoparticles can be occurred. As we all know, the size of Au nanoparticles plays an important role in the coupling between Au nanoparticles. When the size of the Au nanoparticle increases (smaller than 100 nm), the intensity of electric field increases^37^. With the increasing of Au ratio, Au nanoparticles become to an Au film stepwise. In the bimetallic core-shell structures, the outermost layer dominates the interaction with light due to that the electromagnetic fields decay exponentially inside metals. Because the less “free electron behavior”^40^ of Au, the silver has a higher plasmonic efficiency and superior electromagnetic enhancement effect. When the Au film formed on the surface of the Ag, the intensity of localized electric field decreased significantly. With the reaction time increasing, Ag@Au petals become to Ag@Au particles owing to the chemical replacement reaction. Between the Ag@Au core-shell nanoparticles, there are many “hot spots” formed which leads to the enhanced electric field. While the outermost metal of the structure is Au, which has lower plasmonic efficiency and electromagnetic enhancement as analyzed above, the enhancement ability is weaker than silver slice.

Combining the LSPR spectra shown above, SEM photos and Ag-Au ratios of flower-like nanostructures, we think the enhancement of Raman signal originated from the localized field enhancement. The localized field enhancement is from two aspect, “hotspots” and the plasmonic mode coupling. The increase of the Au ratio is not the crucial reason for the enhanced SERS effect. The surface morphology and shape of the Ag-Au hetero-structure play important roles in SERS which is consistent with the results reported previous^15^.

### Chemical Stability of flower-like nanostructures

**Figure 7:**
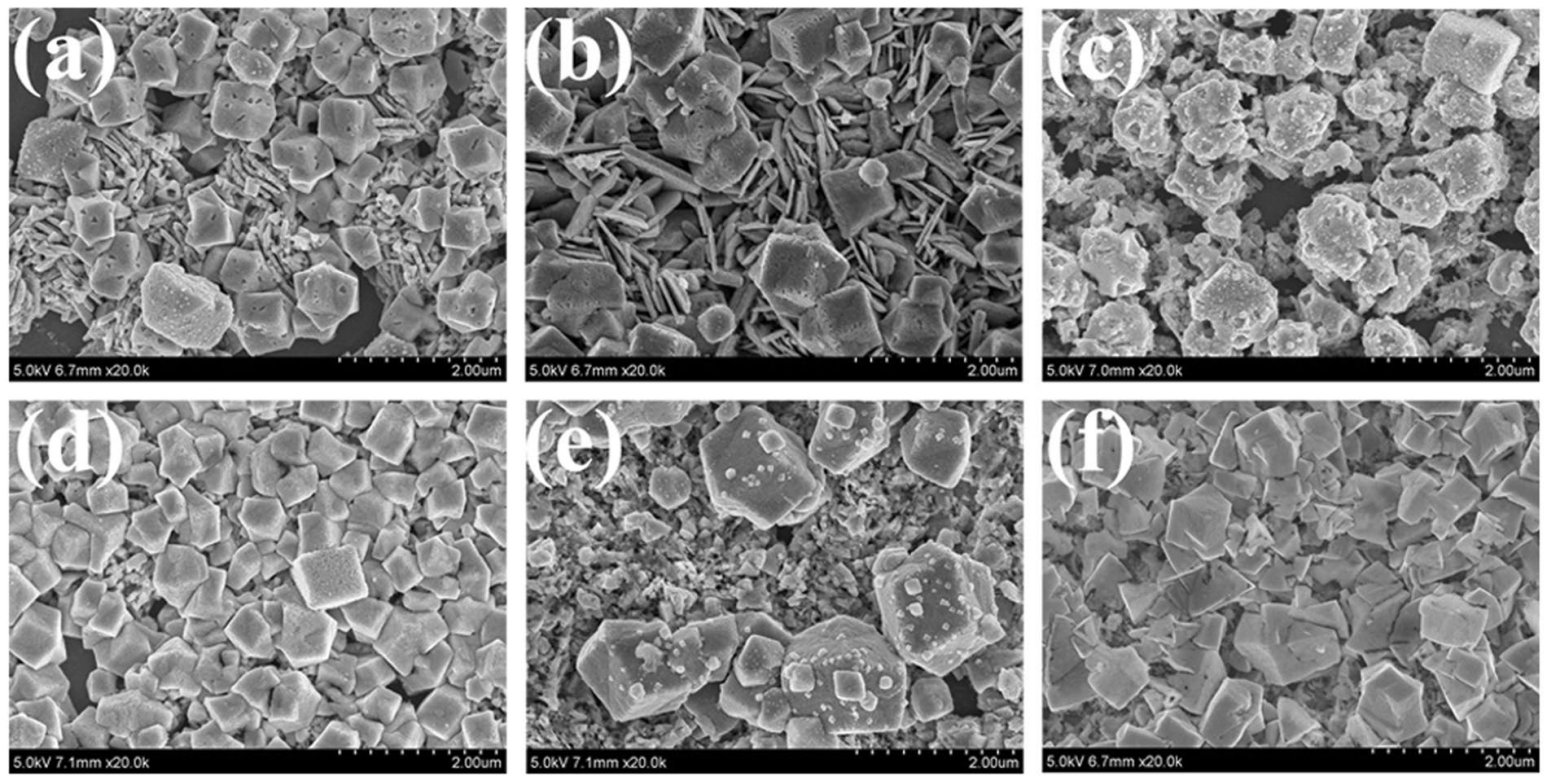
The FESEM pictures of Ag-Au hetero-nanostructures and flower-like Ag nanostructures as a function of time reaction with H_2_O_2_. (**a–c**) Are the FESEM pictures of the flower-like Ag-Au hetero-nanostructures after etching for 5 h, 10 h and 24 h by H_2_O_2_, respectively. (**d–f**) Are the FESEM pictures of the flower-like Ag nanostructures after etching for 5 h, 10 h and 24 h by H_2_O_2_, respectively.

In order to evaluate the chemical stability of the flower-like Ag nanostructures and flower-like Ag-Au hetero-nanostructures, we performed a series of experiments according to the method from the work of Dong Qin’s group^15^. We took the flower-like silver nanostructures and flower-like Ag-Au hetero-nanostructures to react with 2.3% H_2_O_2_ solution for different times up to 24 h, and recorded the microscopic pictures and LSPR spectra as a function of time to monitor the changes in nanoscale. From the spectra (Figure S1 and Figure S2 shown in Supplementary Information), we can see that the spectra intensity almost remained unchanged of the Ag nanostructures and Ag-Au hetero-nanostructures after H_2_O_2_ etching for 0.5 h to 2 h with the little changed shapes. When the etching time is longer than 2 h, the intensities of the LSPR peaks drop quickly. When the etching time is up to 24 h, the intensity of LSPR spectrum of the Ag-Au hetero-nanostructures becomes to 38% of spectrum before etching while the spectrum of Ag nanostructures comes to 10%. Comparing with the results reported before, the silver nanocubes showed a very poor chemical stability in H_2_O_2_ with a complete drop in intensity for the LSPR peak within 3 min^15^, the chemical stability of flower-like Ag-Au nanostructures and flower-like Ag nanostructures are better. However, the Ag-Au hetero-nanostructures are better than flower-like Ag nanostructures in chemical stability. In order to verify the result further, Fig. 7 shows the microscopic pictures obtained by FESEM. Figure 7(a–c) show the FESEM pictures of the Ag-Au hetero-nanostructures after etching for 5 h, 10 h and 24 h by H_2_O_2_. Figure 7(d–f) show the FESEM pictures of the flower-like Ag nanostructures after etching for 5 h, 10 h and 24 h by H_2_O_2_. When the reaction time is 10 h, there are still many flower-like structures can be observed after H_2_O_2_ etching (Fig. 7(b)). While there are no flower-like nanostructures can be identified on the flower-like Ag nanostructures after reacting for 5 h (Fig. 7(d)). The SERS enhancement factor is about 10^4^ with the flower-like Ag-Au hetero-nanostructures after etching for 24 h while the flower-like Ag nanostructure is almost zero. From the results above, we can conclude that the chemical stability of the Ag-Au hetero-nanostructures is improved compared with flower-like Ag nanostructures.

## Conclusions

In this work, we have demonstrated the highly effective surface enhanced Raman scattering substrate with flower-like 3D Ag-Au hetero-nanostructures fabricated by electrodeposition in liquid crystalline phase and galvanic replacement reaction. Flower-like 3D Ag-Au hetero-nanostructures with different morphologies were synthesized and investigated by experiments. The best enhancement factor of Ag-Au hetero-nanostructure substrate is about 8.6 × 10^7^. The reasons that surface morphology and shape of the flower-like Ag-Au hetero-nanoparticles play significant roles in enhancing SERS were discussed by analyzing the results of LSPR spectra, SEM photos and Ag-Au ratio of the different Ag-Au nanostructures. Experimental results show that the chemical stability of Ag-Au hetero-nanostructures is improved compared with the flower-like silver nanostructures. Considering the ultra-sensitive SERS effect, improved chemical stability together with the simple fabrication method and low cost property, the flower-like 3D Ag-Au hetero-nanostructures fabricated by electrodeposition in liquid crystal template and galvanic replacement reaction are good candidates for potential applications in SERS detection and imaging.

## Methods

### Preparation of flower-like silver nanostructures

The flower-like silver nanostructures were fabricated by electrodeposition in the liquid crystalline phase soft template. The liquid crystalline phase used in this work consists of the anionic surfactant sodium bis (2-ethylhexyl) sulfosuccinate (AOT) (97 wt%), the oil phase p-xylene (99 wt%) and water which was replaced by AgNO_3_ aqueous solution in this work. At first, the AOT was dissolved in the p-xylene solution with 1.4 M concentration. Then, 0.3 M AgNO_3_ aqueous solution was added to the mixture drop by drop. After 2 h violent stirring, the mixture became to clear liquid which was used in electrodeposition process as electrolyte. In the electrodeposition process, the silver foil (the anode) was mounted with the ITO glass (the cathode, 15 × 30 mm^2^) to form a cell with 0.7 mm cell gap. The surface of ITO glass was very clean and smooth for collecting the flower-like silver nanostructures. The 3.0 V DC voltage was applied between the anode and cathode by a DC voltage-stabilized power supply at room temperature (~22 °C). When the deposition process was finished, the negative electrode ITO glass was softly washed by ethanol and dried by a gentle flow of N_2_.

### Synthesis of flower-like Ag-Au nanostructures

HAuCl_4_ solution with 0.05 M and 0.5 M concentration was dropped on different silver flower substrates with 5 h deposition time obtained by the method mentioned above. The galvanic replacement reaction between elemental Ag and Au was proceeded. The reaction was continued for 10 min and 30 min both with 0.05 M and 0.5 M HAuCl_4_ solutions, respectively. The flower-like Ag-Au nanostructures were obtained after washing the substrates with deionized water.

### Instrument and Characterization

The morphology was observed by a field emission scanning electron microscope S-4800 from HITACHI. The chemical constituent of the flower-like silver nanostructures was characterized by the energy dispersive spectrum. LSPR spectra of the flower-like nanostructures were obtained from the Perkin Elmer Lambda 950 spectrometer with an integrating sphere.

### Surface enhanced Raman Scattering Measurements

The flower-like Ag nanostructures and flower-like Ag-Au nanostructures were immerged in 50 μM aqueous solution of 4-MBA overnight. Then, the nanostructures were washed with deionized water. 3 μL aqueous solution of 4-MBA (500 mM) was dropped on a silicon wafer as reference sample. The Raman spectra were recorded using an Ocean Optics QE 65 Pro spectrometer. An InPhotonics 785 nm Raman fiber optics probe was used for excitation and collection, with a 105 μm excitation fiber and a 200 μm collection fiber. The numerical aperture was 0.22. The accumulation times were 1 s for the reference samples and 0.1 s for the SERS samples. For each sample, we took three SERS spectra in different positions of the substrate.

## Electronic supplementary material

Supplementary Information
