# Supplementary material for: Highly effective and chemically stable surface enhanced Raman scattering substrates with flower-like 3D Ag-Au hetero-nanostructures
Source: Sci Rep. 2018 Jan 17;8:898. doi: 10.1038/s41598-018-19165-9 (PMC5772549; doi:10.1038/s41598-018-19165-9)
Supplement: Supplementary file 1 — Supplementary Information [file 41598_2018_19165_MOESM1_ESM.pdf]

Highly effective and chemically stable surface enhanced Raman scattering substrates with flower-like 3D Ag-Au hetero -nanostructures

Ying Zhang[1,3], Chengliang Yang[1,*], Bin Xue[2], Zenghui Peng[1], Zhaoliang Cao[1], Quanquan Mu[1], Li Xuan[1]

[1]*State Key Laboratory of Applied Optics, Changchun Institute of Optics, Fine Mechanics and Physics, Chinese Academy of Sciences, Changchun, Jilin 130033, China*

[2]*State Key Laboratory of Luminescence and Applications, Changchun Institute of Optics, Fine Mechanics and Physics, Chinese Academy of Sciences, Changchun, Jilin 130033, China*

[3]*University of Chinese Academy of Sciences, Beijing 100049, China*

**Corresponding author:* *ycldahai@ciomp.ac.cn*

Calculations of EF were shown below:

For the EF of Ag flower-like nanostructures and Ag-Au flower-like nanostructures, we estimated them in a quantitative way,

$$EF = \frac{I_{SERS}}{I_{Raman}} \times \frac{N_{Raman}}{N_{SERS}} \approx \frac{I_{SERS}}{I_{Raman}} \times \frac{\sigma_s A}{\rho_s fA} \quad \textbf{(1)}$$

where σ_s is the area density of molecules on the substrates for normal Raman detection, A is focal area, ρ_s is the surface density of molecules on the Ag or Ag-Au flower-like nanostructures, f is occupied factor of Ag or Ag-Au flower-like nanostructures on the substrates. The area absorbed by 4-MBA on Ag approximate to 0.25, 0.5 and 0.7 for 0.5 h (Figure 1 (a)), 2 h (Figure 1 (b)) and 5 h (Figure1(c)), respectively. The area absorbed by 4-MBA on flower-like Ag-Au nanostructure was approximate to 0.7.

The area density of molecules on the Ag or Ag-Au flower-like nanostructures was estimated as

follows:

3 μL 500 mM 4-MBA deposited on the silicon wafer, forming a shape of disk with diameter ~ 3 mm.

$$\sigma_s = \frac{3\times10^{-6}\times500\times10^{-3}\times6.02\times10^{23}}{3.14\times(1.5\times10^{-3})^2} m^{-2} = 1.278\times10^{23} m^{-2} \quad \textbf{(2)}$$

The surface density of molecules on the Ag or Ag-Au flower-like nanostructures was estimated as follows: we presumed that molecular footprint of 4-MBA molecules adsorbed as a monolayer was 0.54 nm^2 [1].

$$\rho_s = \frac{1}{0.54\times10^{-9}\times10^{-9}} m^{-2} = 1.852\times10^{18} m^{-2} \quad \textbf{(3)}$$

We set the EF calculation for silver nanostructures obtained for 5 h as an example:

We assume that the area of silver flower nanostructures occupied 70 % of the detection area, so occupied factor: *f*=0.7. For SERS experiment, accumulation time was 0.1 s, while for normal Raman detection, accumulation time was 1 s.

$$EF = \frac{2210/0.1}{186}\times\frac{1.278\times10^{23}}{0.7\times1.852\times10^{18}} = 1.17\times10^{7} \quad \textbf{(4)}$$

The other SERS EFs for silver nanostructure substrates and Ag-Au nanostructure substrates were calculated as above.

Additional Figures:

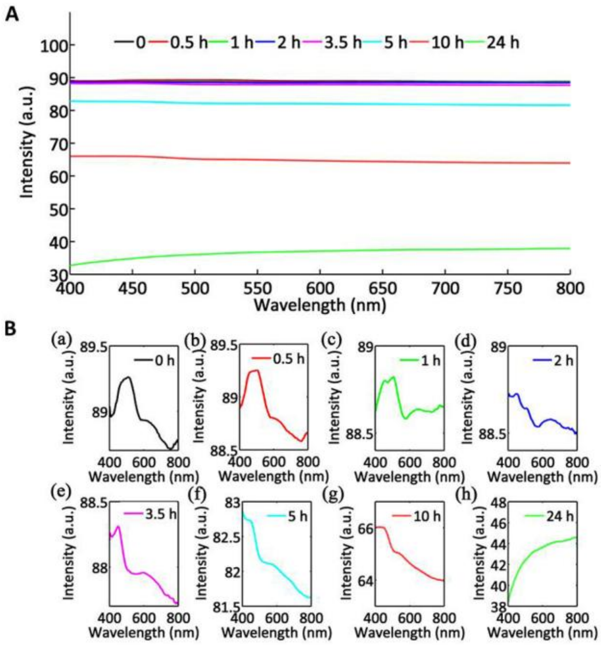

Supplementary Figure S1. LSPR spectra of Ag-Au hetero- nanostructures after etching by H_2O_2 for different times. A shows the LSPR spectra of Ag-Au hetero-nanostructures reacted for 0 h, 0.5 h, 1h, 2h, 3.5 h, 5h, 10h and

24 h in the same coordinate system. **B (a-h)** are the LSPR spectra of Ag-Au hetero nanostructures after etching by H_2O_2 for 0 h, 0.5 h, 1h, 2h, 3.5 h, 5h, 10h and 24 h, respectively.

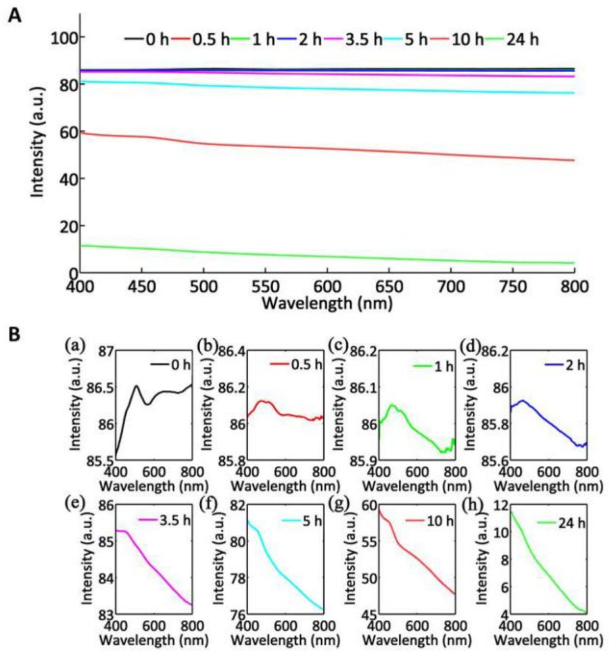

Supplementary Figure S2. LSPR spectra of flower-like Ag nanostructures after etching by H_2O_2 for different times. A shows the LSPR spectra of flower-like Ag nanostructures reacted for 0 h, 0.5 h, 1h, 2h, 3.5 h, 5h, 10h and 24 h in the same coordinate system. **B (a-h)** are the LSPR spectra of flower-like Ag nanostructures after etching by H_2O_2 for 0 h, 0.5 h, 1h, 2h, 3.5 h, 5h, 10h and 24 h, respectively.

References:

1. Xia, X. *et al.* Silver Nanocrystals with Concave Surfaces and Their Optical and Surface-Enhanced Raman Scattering Properties. *Angew. Chem. Int. Edit.* **50,** 12542-12546 (2011).
